# Effect of β-Caryophyllene on insulin resistance in skeletal muscle of high fat diet and fructose-induced type-2 diabetic rats

**DOI:** 10.6026/97320630017741

**Published:** 2021-08-31

**Authors:** Vadivel Mani, Ramya Badrachalam, Sharon Nallathannikulam Shanmugam, Manikandan Balraj, Revathi Kasthuri, Anandhi Danavel, Shyamaladevi Babu

**Affiliations:** 1 Department of Biochemistry, Arunai Medical College & Hospital, Tiruvanamallai-606603, Tamilnadu, India;; 2 Department of biochemistry, MeenakshiAmmal Dental College & Hospital, Chennai-600095, Tamilnadu, India; 3 Department of Physiology, Konaseema Institute of Medical sciences and research foundation, Amalapuram, East Godavari Dt-533201, Andhra Pradesh, India; 4 Department of Research, Meenakshi Academy of Higher Education, Chennai-600078, Tamilnadu, India; 5 Department of Biochemistry,Saveetha Dental College and Hospitals, Saveetha Institute of Medical and Technical Sciences, Saveetha University, Chennai, Tamil Nadu, India

**Keywords:** β-Caryophyllene, High fat diet, Insulin resistance, Type-2 diabetes, IRS-1/Akt signaling, Glucose transporter

## Abstract

High fat diet feeding results in hyperglycemia and insulin resistance, which is a major pathological feature of type-2 diabetes mellitus. The use of oral hypoglycaemic drugs is limited due to its deleterious side effects and there is a need to find more efficacious agents for diabetes management. Hence, it is of interest to show the mechanism of action of β-Caryophyllene on insulin signalling molecules in gastrocnemius muscle of high fat diet - induced type-2 diabetic rats. An oral effective dose of with β-Caryophyllene (200 mg/kg b.wt) was given for 30 days to high fat diet (comprising 2% cholesterol, 1% cholic acid, 30% coconut oil, 67% conventional rat feed) and fructose fed type-2 diabetic rats to find out whether β-Caryophyllene regulates IRS-1/Akt pathway of insulin signalling. The data shows that, β-Caryophyllene treatment significantly increased the mRNA and protein expression of insulin receptor (IR) in diabetic rats whereas there is no significant difference in mRNA expression of insulin receptor-substrate-1 (IRS-1) was observed among groups. The Akt mRNAand GLUT-4mRNA and protein level were also improved in gastrocnemius muscle of type-2 diabetic rats. Thus, we concluded that β-Caryophyllene could be used as potential phyto medicine for type-2 diabetes management.

## Background:

Type-2 diabetes is a progressive condition in which the body grows resistant to the usual impact of insulin and/or gradually loses the ability of the pancreas to produce enough insulin [1]. Western-style diets, low in dietary fiber and high in saturated fatty acids, are implicated in increased risk of diabetes and obesity [2]. The predominance of insulin insensitivity, a more vital patho physiological parameter that contributes to the development of T2DM and an independent risk factor for the metabolic syndrome and much more generalized [3]. A cardinal mechanism for the sustainability of glucose homeostasis is the rapid action of insulin to stimulate the uptake and metabolism of glucose in tissues [4]. Skeletal muscle and liver were the main sites of glucose elimination in the insulin-stimulated state and it was recommended that it be the main tissue responsible for postprandial hyperglycemia in an insulin resistant subject [5]. High fat induced several complications in insulin signaling molecule and cause the insulin insensitivity [6]. The use of natural phytochemicals like β-Caryophyllene to treat the insulin resistance is shown [7].

β-Caryophyllene is a natural sequiterpene, widely present in cannabis as well as many culinary herbs and spices. Black pepper, cloves, cinnamon, hops, rosemary and hemp are good sources of this terpene. It has a many biological effects such as antioxidant, anti-inflammatory and anti-lipidemic effects [8]. Chronic oral administration of β-Caryophyllene reduces glyceamia, depressive-like behavior and neuropathic pain in streptozotocin (STZ)-induced diabetic mice [9]. In addition, recently, it has been elucidated that β-Caryophyllene effectively protects β-cells by alleviating hyperglycemia through increasing insulin release, and also ameliorate oxidative stress and inflammation in pancreatic tissue of experimental diabetic rats [10]. Therefore, it is of interest to show the effect of β-Caryophyllene on insulin resistance in skeletal muscle of high fat diet and fructose-induced type-2 diabetic rats.

## Materials & Methods:

### Chemicals:

All chemicals and reagents used in this investigation were obtained from Sigma Chemical Company (St. Louis, MO, USA); Invitrogen (USA); Eurofins Genomics India Pvt Ltd (Bangalore, India); New England Biolabs (NEB) (USA); Promega (USA); Santa Cruz Biotechnology (USA) and Cell Signaling Technology (USA).β-actin monoclonal antibody was bought from Sigma (USA). Total RNA isolation reagent (TRIR) was obtained from Invitrogen, USA. The reverse-transcriptase enzyme was boughtfrom New England Biolabs (NEB) (USA) and Go Taq Green master mix was obtained from Promega (USA). Insulin receptor (IR), insulin receptor substrate-1 (IRS-1), Akt, glucose transporter-4 (GLUT4) and β-actin primers were purchased from Eurofins Genomics India Pvt Ltd (Bangalore, India) and Polyclonal IR and GLUT4 antibodies were purchased from Santa Cruz Biotechnology, Inc. (Santa Cruz, C.A). 

### Animals:

In our investigation, 150-180 day old Wistar strain healthy adult male albino rats were employed. They were cared for in accordance with national norms and protocols authorised by the Institutional Animal Ethics committee (IAEC No: 007/2019, dated 04/11/2019) at Meenakshi Medical College and Research Institute, MAHER, Enathur, Kanchipuram, Tamil Nadu-631552, India. Animals were kept at a particular temperature (21 ± 2°C) and humidity (65 ± 5 %), with a consistent 12 h light and 12 h dark cycle, and fed a standard pelleted diet (Lipton India, Mumbai, India), with clean drinking water available ad libitum.

### Induction of Type-2 Diabetes:

Rats were made diabetic (type-2) by feeding them a high fat diet comprising 2% cholesterol, 1% cholic acid, 30% coconut oil, 67% conventional rat feed, and 25% fructose via drinking water for 60 days [11]. After 60 days, fasting blood glucose levels were measured, and animals with blood glucose levels more than 120 mg/dl were chosen for the experiment. The high fat diet and sugar feeding were kept up until the end of the research. Normal pelleted rat feed was supplied to the control rats, and water was provided ad libitum.

### Experimental design:

The following experimental design was framed and accordingly the rats were subjected to treatment for a period of one month. Healthy adult male Wistar rats were divided into the following groups of 6 rats each. Control and experimental animals were given an oral glucose tolerance test (OGTT) and an insulin tolerance test (ITT) two days before they were sacrificed. After 30 days, blood was drawn and the animals were perfused with physiological saline while anaesthetized with sodium thiopentone (40 mg/kg b.wt) and skeletal muscle was dissected out to evaluate various properties.

### mRNA expression analysis:

Total RNA Isolation, cDNA conversion and real-time PCR:

Total RNA was extracted from control and experimental samples using a TRIR kit (Total RNA Isolation Reagent Invitrogen). In brief, 1 ml of TRIR was added to 100 mg fresh tissue and homogenized. The contents were immediately transferred to a microcentrifuge tube, where they were mixed with 0.2 ml of chloroform, vortexed for 1 minute, and kept at 4°C for 5 minutes. The contents were then centrifuged for 15 minutes at 4°C at 12,000g. The top layer of the aqueous phase was carefully transferred to a new microfuge tube, and an equal amount of isopropanol was added, vortexed for 15 seconds, and then put on ice for 10 minutes. The supernatant was separated after centrifugation of the content at 12000g for 10 minutes at 4°C. The vortex was used to wash the RNA pellet in 1 ml of 75% ethanol. Using Fourney's et al. [12 - see PDF] method, the isolated RNA was spectrometrically estimated. The amount of RNA in each sample was measured in micrograms. Complementary DNA (cDNA) was synthesized from 2 micrograms of total RNA according to the manufacturer's protocol using a reverse transcriptase kit from Eurogentec (Seraing, Belgium). To perform real-time PCR, a 45 µl reaction mixture including 2x reaction buffer (Takara SyBr green master mix), forward and reverse primers for the target and housekeeping genes, water and β-actin (primer sequences are provided in (Table 1 - see PDF) was prepared. In individual PCR vials, about 5 µl of control DNA for positive control, 5 µl of water for negative control and 5 µl of template cDNA for samples were taken and reaction mixture (45 µl) was added. 40 cycles (95°C for 5 min, 95°C for 5 s, 60°C for 20 s and 72°C for 40 s) was set up for the reaction. Results were plotted using the PCR machine (Stratagene MX 3000P, Agilent Technologies, 530l, Stevens Creek Blvd, Santa Clara CA, 95051). Relative quantification was calculated from the melt and amplification curves analysis.

### Protein expression analysis:

Protein isolation and western blotting:

100 mg of gastrocnemius muscle from control and experimental animals were used to isolate proteins. 1 ml of buffer A (5 mM NaN3, 0.25 M sucrose, 10 mM NaHCO3) was added to 100 mg of gastrocnemius muscle, homogenised, and centrifuged at 1300xg at 4°C for 10 minutes. The supernatant was separated and centrifuged at 12,000xg for 15 minutes at 4°C. To evaluate the post-receptor insulin signaling molecules, the final supernatant was sampled as a total protein. The protein estimation was done using the Lowry et al. [18] technique.

The lysate proteins (50g/lane) were isolated and electro blotted onto a polyvinylidenedifluoride (PVDF) membrane (Bio-Rad Laboratories Inc) using sodium dodecyl sulfate-polyacrylamide gel electrophoresis (10 % gel). The membranes were blocked with 5% non-fat dry milk and tagged with primary antibodies (1:1000 dilutions). After three washes with TBS-T, the membrane was incubated for 1 hour with a 1:5000 dilution of horseradish peroxidase-conjugated rabbit-anti-mouse or goat-anti-rabbit secondary antibody (GeNei, Bangalore, India). Following the incubation period, the membrane was washed three times with TBS and TBS-T. The protein bands were visualised using a sophisticated Chemiluminescence detection system (Thermo Fisher Scientific Inc., Waltham, MA, USA), the specific signals were found, and protein bands were captured and quantified using Chemidoc and Quantity One image analysis systems from Bio-Rad Laboratories, CA. The membrane was then stripped for 30 minutes at 50°C in stripping buffer (50 ml, 62.5 mMTris-HCl (pH 6.7), 1 g SDS, and 0.34 ml -mercaptoethanol). The membranes were then reprobed using an anti β -actin antibody (1:5000). The invariant control used was β-actin.

### Statistical analysis:

Using one-way analysis of variance (ANOVA) and Duncan's multiple range test, computer-based software, the data were analyzed to determine the significance of individual variance within the control and treated groups (Graph Pad Prism version 5). Duncan's test was used to determine significance at the level of p<0.05.

## Results and Discussion:

In the wake of the worldwide increase in the prevalence of type-2 diabetes Mellitus, intensive focus of research is development of new drugs and understanding the signalling pathways impacting these diseases, to treat diabetes mellitus and its complications. Insulin signalling plays a vital role in the control of a wide range of biological process such as glucose, lipid, and energy homeostasis, predominantly via action on liver, skeletal muscle, and adipose tissue. Disturbances in these signalling pathways can lead to insulin resistance. Insulin resistance is identified as major pathological feature of type-2 diabetes mellitus and it is presumed to disturb the carbohydrate, protein and lipid metabolism resulting in sustained hyperglycemia [19]. Many researches has been documented that high fat diet feeding results in hyperglycemia, hyperinsulinemia, hyperlipidemia and insulin resistance mice [20 - see PDF]. In the present study we attempts to elucidate the mechanism of action of β-Caryophyllene during insulin resistance through in vivo approaches with an objective to understand whether β-Caryophyllene improves the gene and protein expression of insulin signalling molecules. We have shown that, β-Caryophyllene, a sequisterpene, restored the altered gene and protein expression of insulin signalling molecules such as IR, IRS-1, Akt and GLUT-4 in skeletal muscle thereby improves insulin sensitivity and signalling in diabetic rats.

**Figure 1:**
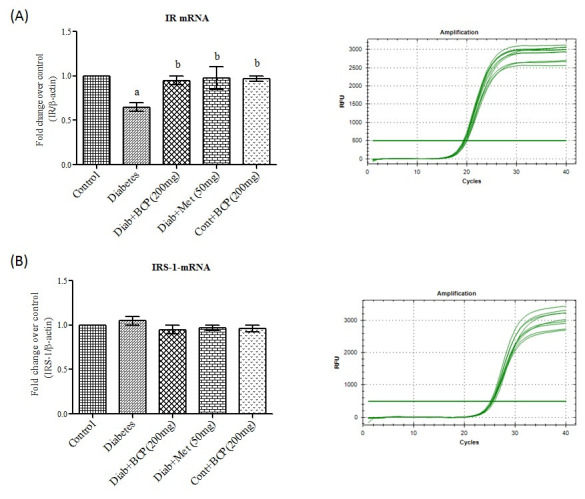
Effect of β-Caryophyllene on IR and IRS-1 mRNA expression in gastrocnemius muscle of type-2 diabetic rats. Each bar represents mean ± SEM (n = 6). The 'F' and 'P' values are by one-way ANOVA with Student NewmanKeul's multiple comparison test. Significance at P< 0.05. a- significantly different from control group. b- significantly different from type-2 diabetic group.

Insulin receptor is a transmembrane signalling protein belongs to the receptor tyrosine kinase (RTK) family, which are present on the surface of target cells such as liver, fat and muscle. These are important regulator of cell growth, differentiation and metabolism. Insulin binding to insulin receptor results tyrosine auto phosphorylation of the β subunit, which phosphorylates other substrates and initiate signaling cascade [21]. In the present study, IR mRNA (Figure 1A) and protein (Figure 3A) levels were found to be decreased in skeletal muscle of high fat and fructose induced type-2 diabetic rats and β-Caryophyllene treatment showed a significantly improved the IR mRNA and protein levels in diabetic rats. It has been reported that IR gene expression is diminished due to increased free fatty acid and contributes to a decline of insulin receptor protein in insulin target tissues [22]. Being a potent antioxidant and anti-hyperlipidemic agent [10], β-Caryophyllene increases notably the IR levels in liver and skeletal muscle by decreasing the lipid levels intype-2 diabetic rats.

As a result of auto phosphorylation of insulin receptor, the substrate molecule of insulin receptor is phosphorylated at tyrosine residue and activated. The insulin receptor substrate (IRS1) is a critical element, plays an important role in the metabolic actions of insulin-signalling pathways, mainly in skeletal muscle and adipose tissue whereas IRS-2 is present in liver. Here, in the present study, we observed that there was a significant difference in IRS-1 mRNA (Figure 1B) among the experimental groups, but high fat diet–induced type-2 diabetic groups showed a significant decrease in mRNA expression levels of IRS-1 in skeletal muscle of high fat diet induced type-2 diabetic rats. It has been reported that high fat diet increases the concentration of intracellular fatty acyl-CoA and DAG, which influence the genes responsible for IR and IRS-1 transcription factors [24]. Experimental rats treated with β-Caryophyllene showed a significant increase in the IRS-1 mRNA in skeletal muscle which may be attributed due to free radical scavenging, anti-hyperlipidiemic and anti-inflammatory activity of β-Caryophyllene.

**Figure 2:**
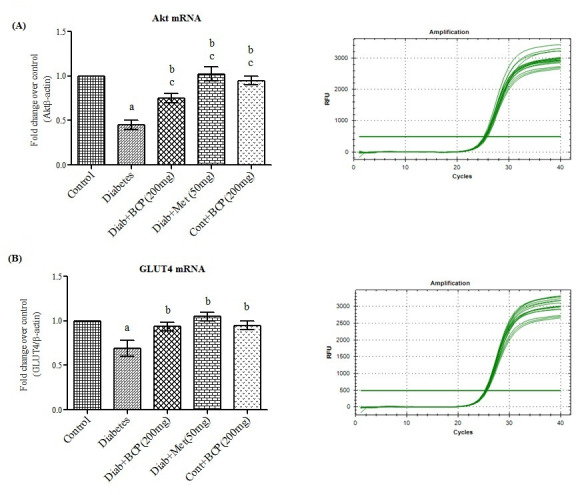
Effect of β-Caryophyllene on Akt and GLUT4 mRNA expression in gastrocnemius muscle of type-2 diabetic rats. Each bar represents mean ± SEM (n = 6). The 'F' and 'P' values are by one-way ANOVA with Student Newman Keul's multiple comparison test. Significance at P< 0.05. a-significantly different from control group. b-significantly different from type-2 diabetic group. c - Significantly different from β-Caryophyllene treated type 2 diabetic rats.

**Figure 3:**
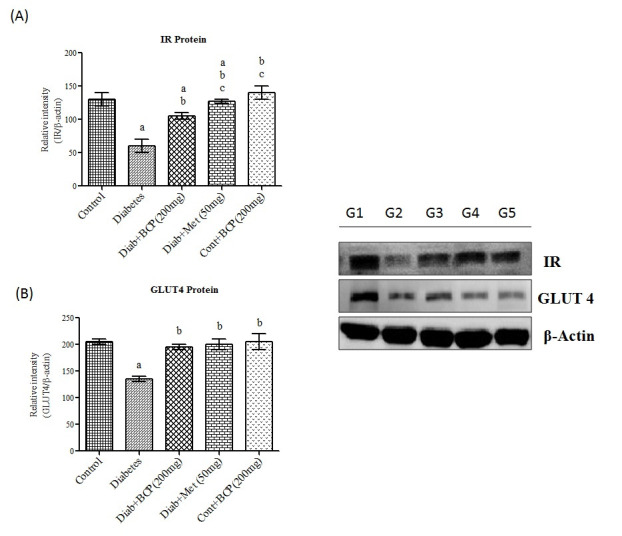
Effect of β-Caryophyllene on IR and GLUT4 Protein expression in gastrocnemius muscle of type-2 diabetic rats. Each bar represents mean ± SEM (n = 6). The 'F' and 'P' values are by one-way ANOVA with Student Newman Keul's multiple comparison test. Significance at P< 0.05. G1-Normal control rats, G2-diabetic rats, G3-diabetic rats treated with β-Caryophyllene, G4-diabetic rats treated with metformin, G5-control rats treated with β-Caryophyllene. a-Significantly different from control group. b-Significantly different from type-2 diabetic group, c - significantly different from β-Caryophyllene treated type 2 diabetic rats.

As a master switch of the cellular signaling pathways, activation of Akt (also known as protein kinase B), a serine-threonine kinase triggers insulin effects on target tissues such as glycogen synthesis and activates the translocation of GLUT4 by phosphorylation of Akt Substrate 160 kDa (AS160) [25]. In the present study the mRNA levels of Akt (Figure 2A) were decreased in diabetic groups where astreatment with β-Caryophyllene significantly enhanced the gene expression of Akt in skeletal muscle of diabetic rats and this may be due to increased IR and IRS-1 tyrosine phosphorylation as a result of improved insulin sensitivity. As stated before Akt, a major component, plays a key role in the insulin-regulated GLUT4 trafficking. Decreased expression of Akt leads to decline in the expression of GLUT4 (Glucose transporter) in skeletal muscle of type-2 diabetic rats [26]. β-Caryophyllene treatment significantly enhanced the gene (Figure 2B) and protein (Figure 3B) expression of GLUT 4 as a result of increased Akt activation in skeletal muscle, which in turn significantly increased the glucose uptake, and oxidation in skeletal muscle of type-2 diabetic rats.

## Conclusion:

The obtained data shows that β-Caryophyllene treatment is beneficial for decreasing the progression & risk of insulin resistance and type-2 diabetes by increasing the IR, IRS-1 Akt and GLUT 4 expression in the skeletal muscle of high fat diet and fructose-induced type-2 diabetic rats.
